# Die Kosten der präoperativen Anämie bei Hüftgelenkrevisionsoperationen

**DOI:** 10.1007/s00101-022-01211-x

**Published:** 2022-11-15

**Authors:** Gerald Vorderwülbecke, Claudia Spies, Christian von Heymann, Jochen Kruppa, Daniel Fürstenau, Lutz Kaufner, Sven Werner, Moritz Höft, Felix Balzer

**Affiliations:** 1 Klinik für Anästhesiologie mit Schwerpunkt operative Intensivmedizin, Charité – Universitätsmedizin Berlin (corporate member of Freie Universität Berlin, Humboldt-Universität zu Berlin, and Berlin Institute of Health), Berlin, Deutschland; 2 Klinik für Anästhesie, Intensivmedizin, Notfallmedizin und Schmerztherapie, Vivantes Klinikum im Friedrichshain, Landsberger Allee 49, 10249 Berlin, Deutschland; 3 Department of Digitalization, Copenhagen Business School, Copenhagen, Dänemark; 4 Institut für Biometrie und Klinische Epidemiologie, Charité – Universitätsmedizin Berlin (corporate member of Freie Universität Berlin, Humboldt-Universität zu Berlin, and Berlin Institute of Health), Berlin, Deutschland; 5 Institut für Medizinische Informatik, Charité – Universitätsmedizin Berlin (corporate member of Freie Universität Berlin, Humboldt-Universität zu Berlin, and Berlin Institute of Health), Campus Charité Mitte, Charitéplatz 1, 10117 Berlin, Deutschland; 6 Geschäftsbereich Unternehmenscontrolling – Klinikcontrolling, Charité – Universitätsmedizin Berlin, Berlin, Deutschland

**Keywords:** Patient Blood Management, Erythrozyten, Transfusion, Erlöse, DRG-System, Patient blood managment, Red blood cells, Transfusion, Revenue, DRG system

## Abstract

**Hintergrund:**

Die Anämie hat eine hohe Prävalenz bei Patienten vor Hüftgelenkrevisionsoperation und ist mit einer erhöhten Komplikationsrate assoziiert. Die vorliegende Arbeit untersucht erstmals den Zusammenhang von Kosten, realen DRG-Erlösen und Falldeckung der präoperativen Anämie bei elektiven Hüftgelenkrevisionsoperationen.

**Methoden:**

Für alle Patienten, die sich von 2010 bis 2017 an 2 Campi der Charité – Universitätsmedizin Berlin einer Hüftgelenkrevisionsoperation unterzogen, wurden Daten zu Patienten sowie Transfusionen, Kosten und Erlösen gesammelt. Subgruppen- und lineare Regressionsanalysen untersuchten die Falldeckung anämischer und nichtanämischer Patienten.

**Ergebnisse:**

Von 1187 eingeschlossenen Patienten waren 354 (29,8 %) präoperativ anämisch. Insgesamt wurden 565 (47,6 %) Patienten, mit einem deutlichen Überwiegen anämischer Patienten (72,6 % vs. 37,0 %, *p* < 0,001), transfundiert. Kosten (12.318 € [9027;20.044 €] vs. 8948 € [7501;11.339 €], *p* < 0,001) und Erlöse (11.788 € [8992;16.298 €] vs. 9611 € [8332;10.719 €], *p* < 0,001) waren für anämische Patienten höher, die Fallkostendeckung defizitär (−1170 € [−4467;1238 €] vs. 591 € [−1441;2103 €] €, *p* < 0,001). Bei anämischen Patienten nahm die Falldeckung mit zunehmender Transfusionsrate ab (*p* ≤ 0,001). Komorbiditäten hatten keinen signifikanten ökonomischen Einfluss.

**Schlussfolgerung:**

Die präoperative Anämie und perioperative Transfusionen bei Hüftgelenkrevisionsoperationen sind mit erhöhten Behandlungskosten und einer finanziellen Unterdeckung für Kostenträger im Gesundheitswesen verbunden. Konzepte zur Behandlung der präoperativen Anämie (z. B. Patient Blood Management) könnten mittelfristig Behandlungskosten senken.

**Zusatzmaterial online:**

Die Online-Version dieses Beitrags (10.1007/s00101-022-01211-x) enthält weitere Tabellen und eine Abbildung.

## Abstract (en)

**Background:**

Anemia is highly prevalent in patients before hip joint revision surgery (HJRS) and is associated with an increased complication rate. This paper is the first to investigate costs, real diagnosis-related group (DRG) revenues and case coverage of preoperative anemia in elective HJRS.

**Methods:**

Medical data, transfusions, costs, and revenues of all patients undergoing HJRS at two campuses of the Charité –Universitätsmedizin Berlin between 2010 and 2017 were used for subgroup analyses and linear regressions.

**Results:**

Of 1187 patients included 354 (29.8%) showed preoperative anemia. A total of 565 (47.6%) patients were transfused with a clear predominance of anemic patients (72.6% vs. 37.0%, *p* < 0.001). Costs (12,318€ [9027;20,044€] vs. 8948€ [7501;11,339€], *p* < 0.001) and revenues (11,788€ [8992;16,298€] vs. 9611€ [8332;10,719€], *p* < 0.001) were higher for preoperatively anemic patients and the coverage was deficient (−1170€ [−4467;1238€] vs. 591€ [−1441;2103€], *p* < 0.001). In anemic patients, case contribution margins decreased with increasing transfusion rates (*p* ≤ 0.001). Comorbidities had no significant economic impact.

**Conclusion:**

Preoperative anemia and perioperative transfusions in HJRS are associated with increased treatment costs and a financial undercoverage for healthcare providers and health insurance companies. Concepts for the treatment of preoperative anemia (e.g. patient blood management) could reduce treatment costs in the medium term.

## Hinführung zum Thema

Der Maßnahmenkatalog „Patient Blood Management“ zum individuellen Ausgleich einer präoperativen Anämie wird in immer mehr Kliniken etabliert. Dennoch wird häufig noch die allogene Transfusion von Erythrozytenkonzentraten (EK) als schnellverfügbare Therapie der akuten und chronischen Anämie genutzt [17]. Während die medizinischen Komplikationen von Anämie und Transfusion gut erforscht sind, fehlen Daten zu finanziellen Aspekten von Anämie und Transfusion in der perioperativen Medizin. Die folgende Arbeit analysiert anhand von Behandlungskosten, DRG-Erlösen und den resultierenden Falldeckungen den ökonomischen Einfluss von präoperativer Anämie und EK-Transfusion.

## Einleitung

Die Anämie ist insbesondere im höheren Lebensalter eine der Erkrankungen mit der höchsten Prävalenz in den industrialisierten Ländern. Gleichzeitig weisen Hüftgelenkersatzoperationen in den letzten Jahren eine deutliche Zunahme auf [26]. Dies hat zur Folge, dass auch die Zahl der Hüftgelenkrevisionsoperationen (HGRO) weiter zunimmt [13]. Dabei weisen Patienten, die sich einer HGRO unterziehen müssen, neben mehr Komorbiditäten insbesondere eine höhere Anämierate auf [11]. Die präoperative Anämie ist bei diesen Patienten mit einem erhöhten perioperativen Komplikations- und Sterberisiko [5], einem verlängerten Krankenhausaufenthalt [15, 16] und einer höheren EK-Transfusion-Rate [25] assoziiert.

Während die medizinischen Komplikationen einer präoperativen Anämie gut untersucht sind [11, 15, 16, 24], liegen für die Kosten, die eine Anämie vor elektiven Operationen verursacht, bislang wenige, v. a. US-amerikanische, Berechnungen vor. Diese belegen konsistent eine Erhöhung der stationären Behandlungskosten gegenüber nichtanämischen Patienten [8, 10, 20]. Demgegenüber wurden für ein australisches Krankenhaus höhere Nettoerlöse durch PBM vor kolorektaler Chirurgie nachgewiesen [21]. Für Deutschland sind bisher Berechnungen der Kosten der präoperativen Anämie hinsichtlich einer Transfusion im Rahmen der Implementierung eines „Patient-Blood-Management“(PBM)-Konzepts auf krankenhaus- bzw. gesellschaftlicher Ebene publiziert worden [6, 12]. Detaillierte Berechnungen, die die Kosten der präoperativen Anämie den realen DRG-Erlösen gegenüberstellen, existieren nach Wissen der Autoren derzeit für den deutschen Krankenhaussektor nicht.

Der Gegenstand der vorliegenden Untersuchung ist daher die Analyse von stationären Behandlungskosten, DRG-Erlösen und der resultierenden Falldeckung von präoperativ anämischen Patienten, die sich einer HGRO unterzogen.

## Methoden

### Datenquellen

Die vorliegende Arbeit ist eine retrospektive Kohortenstudie aus der Charité – Universitätsmedizin Berlin. Das Votum der Ethikkommission (EA1/343/16) wurde vor Erstellung der Datenbank und statistischer Analyse eingeholt. Aufgrund des Studiendesigns entfiel der Bedarf für eine Patienteneinwilligung. Routinedaten aller Patienten, die sich von 2010 bis einschließlich 2017 an den Campi Charité Campus Mitte und Charité Campus Virchow-Klinikum einer operativen Endoprothesenrevision des Hüftgelenks unterzogen hatten, wurden aus 3 elektronischen Patientendatenmanagementsystemen erhoben (COPRA System GmbH, Sasbachwalden, MEDLINQ Softwaresysteme GmbH, Hamburg, und SAP AG, Walldorf). Der Zeitraum wurde gewählt, um ein ausreichend aussagekräftiges Patientenkollektiv zu generieren und gleichzeitig Änderungen in den Operationsprozeduren möglichst niedrig zu halten.

Für die identifizierten Fälle stellte das Klinikcontrolling Kostendaten zur Verfügung. Dabei handelte es sich bei jedem Patienten um die Kosten des gesamten stationären Behandlungsfalles sowie des auf Grundlage der DRG-Einordnung erwirtschafteten Erlöses. Die Falldeckung errechnete sich als Differenz von Erlös und Kosten.

### Studienpopulation

Eingeschlossen wurden alle Patienten, für die im Untersuchungszeitraum einer der OPS-Codes 5‑820 (*Implantation einer Endoprothese am Hüftgelenk*) und 5‑821 (*Revision, Wechsel und Entfernung einer Endoprothese am Hüftgelenk*) dokumentiert war. Im Falle des Codes 5‑820 war auch die ICD-10-Diagnose T84 (*Komplikationen durch orthopädische Endoprothesen, Implantate oder Transplantate*) erforderlich, um Ersteingriffe auszuschließen. Bei wiederholt operierten Patienten wurde nur die erste Revisionsoperation berücksichtigt, um eine Verfälschung der Ergebnisse durch mehrfachen Einschluss identischer Patienten zu verhindern. Datensätze von Patienten ohne eine präoperative Hämoglobinmessung oder mit unvollständigen Kostendaten wurden ausgeschlossen, ebenso Fälle, in denen die Ursache der Revision weder als mechanisch noch infektiös kategorisiert worden war, um die Unterscheidung zwischen septischer und aseptischer Indikation als möglichem Confounder berücksichtigen zu können.

### Definitionen

#### Anämie

Die binäre Einteilung nach Anämie bei Aufnahme basierte auf dem ersten bei stationärer Aufnahme gemessenen Hämoglobinwert und erfolgte gemäß WHO-Kriterien (Hb < 12 g/dl für Frauen bzw. 13 g/dl für Männer) [4].

#### Intra- vs. perioperative Transfusion

Die ermittelte Anzahl der pro Patient verabreichten EK basiert auf der Datenbank des Zentrums für Transfusionsmedizin und Zelltherapie der Charité. Um den Einfluss von akutem Blutverlust als Transfusionstrigger zu minimieren, wurde angenommen, dass perioperative, also prä- und/oder postoperativ verabreichte, Transfusionen dem Ausgleich einer chronischen Anämie dienten und intraoperative Transfusionen primär zum Ausgleich eines intraoperativ auftretenden Blutverlusts verabreicht wurden. Da die Indikation zur intraoperativen Transfusion retrospektiv aber nie exakt zwischen präoperativer Anämie und intraoperativem Blutverlust trennen kann, wurde ebenfalls die Berechnung unter Einschluss der intraoperativen Transfusionen durchgeführt (Zusatzmaterial online, Box am Anfang des Artikels). Die Unterscheidung bezüglich des Transfusionszeitpunktes erfolgte anhand der Narkoseprotokolle, welche entweder elektronisch oder in Papierform vorlagen.

Die Transfusion von Blutprodukten außer EK, z. B. Frischplasma oder Thrombozytenkonzentrate, wurde für die Datenbankanalyse und Ergebnisinterpretation nicht berücksichtigt, da diese nicht zur Therapie einer präoperativen Anämie gehören.

### Statistische Analysen

Eine Prüfung des Datensatzes auf Normalverteilung wurde mit dem Shapiro-Wilk-Test vorgenommen. Normal verteilte Ergebnisse werden als Mittelwert ± Standardabweichung, nicht normal verteilte Ergebnisse als Median und Quartile sowie qualitative Ergebnisse als Häufigkeiten und Prozentanteile dargestellt. Für normal verteilte Größen wurde ein zweiseitiger *t*-Test, für nicht normal verteilte der exakte nonparametrische Kruskal-Wallis-Test zur univariaten Analyse der Unterschiede zwischen den Gruppen genutzt. Das Signifikanzniveau betrug *p* < 0,05.

Mit unterschiedlichen linearen Regressionsmodellen wurde der Zusammenhang zwischen Endpunkten und Basischarakteristika untersucht. Die Modellauswahl erfolgte anhand einer schrittweisen Rückwärtsselektion unter Nutzung des Akaike-Informationskriteriums (AIC).

In einem weiteren Schritt wurde für die abhängigen Variablen Kosten, Erlös und Deckung jeweils ein Regressionsmodell mit den unabhängigen Variablen Anämie bei Aufnahme, Anzahl der perioperativ transfundierten EK und dem Produkt der beiden als Interaktionsterm berechnet, um so ggf. einen Moderatoreffekt zu detektieren. Dieser entsteht, wenn eine unabhängige Variable, hier also die präoperative Anämie, den Effekt mindestens einer anderen unabhängigen (der Anzahl der transfundierten EK) auf eine abhängige Variable (Kosten, Erlös bzw. Deckung) beeinflusst [23]. Bei einer solchen Berechnung mit Interaktionsterm sind die Zahlenwerte der Haupteffekte nur begrenzt interpretierbar; anhand des *p*-Wertes lässt sich jedoch eine Aussage treffen, ob eine signifikante Interaktion vorliegt.

Um den Effekt der Transfusion unabhängig von der Anzahl der transfundierten Einheiten zu erfassen, wurde im deskriptiven Gruppenvergleich das Vorliegen einer perioperativen Transfusion als binäre Variable behandelt. In die lineare Regression hingegen floss für eine höhere Aussagekraft die genaue Anzahl der perioperativ transfundierten Einheiten an EK ein.

## Ergebnisse

### Patientencharakteristika

Nach Ausschluss von operativen Prozeduren, die nicht den oben genannten Einschlusskriterien entsprachen, verblieben 1187 vollständig dokumentierte Behandlungsfälle (729 Frauen, 458 Männer). Das mediane Alter bei Aufnahme betrug 71,0 Jahre. Bei 354 (29,8 %) der eingeschlossenen Patienten lag zum Zeitpunkt der Aufnahme eine präoperative Anämie vor. Die Basischarakteristika dieser Kohorte unterschieden sich deutlich von der präoperativ nichtanämischen Gruppe (Tab. 1): Der Frauenanteil war höher (und lag dabei in beiden Gruppen bei über der Hälfte), die Patienten waren im Mittel älter, hatten häufiger einen ASA-Score von 3 oder höher und eine infektiöse Genese ihrer HGRO. Ferner wiesen diese Patienten eine höhere Zahl an Komorbiditäten auf.

**Table 1:** 

	Alle	Keine Anämie	Anämie	*p*-Wert
	*n* = 1187	*n* = 833	*n* = 354	
Alter	71,0 [61,0;77,0]	70,0 [60,0;76,0]	74,0 [65,2;79,0]	< 0,001
Weibliches Geschlecht	729 (61,4 %)	543 (65,2 %)	186 (52,5 %)	< 0,001
Hb bei Aufnahme in g/dl	13,3 [12,0;14,2]	13,8 [13,2;14,6]	11,1 [10,0;11,8]	< 0,001
ASA > 2	471 (40,6 %)	260 (32,0 %)	211 (60,6 %)	< 0,001
Mechanische Genese der Hüftkomplikation	831 (70,0 %)	673 (80,8 %)	158 (44,6 %)	< 0,001
Diabetes	205 (17,3 %)	110 (13,2 %)	95 (26,8 %)	< 0,001
Adipositas	144 (12,1 %)	91 (10,9 %)	53 (15,0 %)	0,063
Hypertonus	748 (63,0 %)	492 (59,1 %)	256 (72,3 %)	< 0,001
Herzinsuffizienz	135 (11,4 %)	71 (8,5 %)	64 (18,1 %)	< 0,001
COPD	49 (4,1 %)	28 (3,4 %)	21 (5,9 %)	0,060
CNV	164 (13,8 %)	70 (8,4 %)	94 (26,6 %)	< 0,001
KHK	146 (12,3 %)	90 (10,8 %)	56 (15,8 %)	0,021
Transfusionen, insgesamt	628 (52,9 %)	337 (40,5 %)	291 (82,2 %)	< 0,001
Perioperative Transfusionen	565 (47,6 %)	308 (37,0 %)	257 (72,6 %)	< 0,001
Anzahl intraoperativer EK	0 [0;0]	0 [0;0]	1 [0;2]	< 0,001
Anzahl perioperativer EK	0 [0;2]	0 [0;2]	2 [0;5]	< 0,001
Gesamtzahl der EK	1 [0;3]	0 [0;2]	3 [2;6]	< 0,001

Mit 565 Patienten (47,6 %) wurde fast die Hälfte aller Patienten außerhalb der Operation transfundiert, mit einer höheren Transfusionsrate der präoperativ anämischen Patienten (257 Patienten (72,6 %) vs. 308 Patienten (37,0 %), *p* < 0,001). Das relative Risiko präoperativ anämischer Patienten, perioperativ eine Bluttransfusion zu erhalten, war annähernd verdoppelt (RR = 1,96; 95 %-KI 1,76–2,19; *p* < 0,001).

### Outcomes

**Figure 1:**
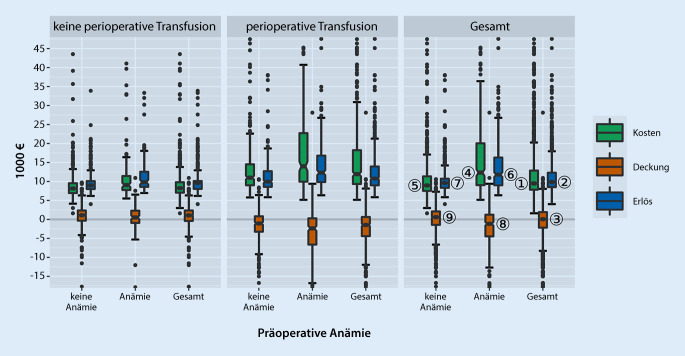

Pro Fall entstanden Kosten in Höhe von 9475 € [7826;12.858] € (in Abb. 1 mit *①* markiert); der Erlös lag bei 9853 € [8514;12.304] € *②*, die Falldeckung aller Patienten bei 68 [−2169,88;1926] € *③.*

Die Gesamtkosten präoperativ anämischer Patienten waren signifikant höher (im Median 12.318 € [9027;20.044] € *④* vs. 8948 € [7501;11.339] *⑤, p* < 0,001), ebenso der Gesamterlös (11.788 [8992;16.298] € ⑥ vs. 9611 [8332;10.719] € ⑦, *p* < 0,001), die Gesamtdeckung dagegen signifikant niedriger (−1170 [−4467;1238] € *⑧* vs. 591 [−1441;2103] € *⑨, p* < 0,001).

Die perioperative Transfusion im Gesamtkollektiv und in den Subgruppen der anämischen bzw. nichtanämischen Patienten (Tab. 2) ging mit signifikant niedrigeren Erlösen, Falldeckungen sowie erhöhten Kosten und verlängerten Liegedauern, bezogen auf den Gesamtaufenthalt und die intensivstationäre Behandlung, einher. Ein vollständiger Gruppenvergleich in Abhängigkeit von der präoperativen Anämie findet sich im Zusatzmaterial online (Box am Anfang des Artikels).

**Table 2:** 

	Gesamt	Keine perioperative Transfusion	Perioperative Transfusion	*p*-Wert
*Alle Patienten*	*n* *=* *1187*	*n* *=* *622*	*n* *=* *565*	–
Kosten [€]	9475 [7826;12.858]	8238 [7043;9797]	11.934 [9332;18.235]	< 0,001
Erlös [€]	9853 [8514;12.304]	9572 [8029;10.109]	10.779 [8992;13.939]	< 0,001
Deckung [€]	68 [−2170;1926]	1060 [−408;2356]	−1464 [−4407;639]	< 0,001
Liegedauer [Tage]	11 [9;15]	10 [9;12]	14 [11;21]	< 0,001
ITS-Dauer [Tage]	0 [0;8]	0 [0;0]	5 [0;19]	< 0,001
*Präoperativ anämische Patienten*	*n* *=* *354*	*n* *=* *97*	*n* *=* *257*	*–*
Kosten [€]	12.318 [9027;20.044]	9049 [7747;11.425]	13.978 [9949;22.734]	< 0,001
Erlös [€]	11.788 [8992;16.298]	9816 [8576;12.546]	12.304 [9850;16.796]	< 0,001
Deckung [€]	−1170 [−4467;1238]	520 [−922;2295]	−2366 [−6631;283]	< 0,001
Liegedauer [Tage]	15 [11;23]	11 [9;14]	16 [12;27]	< 0,001
ITS-Dauer [Tage]	4 [0;21]	0 [0;4]	8 [0;29]	< 0,001
*Präoperativ nichtanämische Patienten*	*n* *=* *833*	*n* *=* *525*	*n* *=* *308*	*–*
Kosten [€]	8948 [7501;11.339]	8152 [6942;9524]	11.005 [8996;14.511]	< 0,001
Erlös [€]	9611 [8332;10.719]	8992 [8002;10.093]	9957 [8576;12.700]	< 0,001
Deckung [€]	591 [−1441;2103]	1098 [−333;2357]	−1056 [−3242;823]	< 0,001
Liegedauer [Tage]	11 [9;14]	10 [9;12]	13 [10;16]	< 0,001
ITS-Dauer [Tage]	0 [0;4]	0 [0;0]	3 [0;15]	< 0,001

In der linearen Regression war die präoperative Anämie signifikant mit höheren Fallkosten (2382 €; 95 %-KI 750–4013 €; *p* = 0,004), höherem Erlös (1473 €; 95 %-KI 78–2868 €; *p* = 0,039) und einer niedrigeren Falldeckung (−995 €; 95 %-KI −1679–−312 €; *p* = 0,004) verbunden (Tab. 3). Das Vorliegen mindestens einer perioperativen Transfusion war im Median mit einer Erhöhung der Fallkosten um 5242 € (95 %-KI 3796–6688 €; *p* < 0,001) und des Erlöses um 2814 € (95 %-KI 1578–4050 €; *p* < 0,001) verbunden; die Falldeckung war mit −2455 € defizitär (95 %-KI −3076–−1833 €; *p* < 0,001).

**Table 3:** 

	Schätzwert	2,5 %	97,5 %	*p*-Wert
*Kosten*				
(Intercept)	7175 €	6152 €	8197 €	< 0,001
ASA > 2	1371 €	−97 €	2839 €	0,067
Anämie bei Aufnahme	2382 €	750 €	4013 €	0,004
Infektion der Hüftgelenkprothese	5452 €	3882 €	7022 €	< 0,001
Diabetes	1526 €	−297 €	3348 €	0,101
CNV	1615 €	−392 €	3623 €	0,115
Perioperative Transfusion	5242 €	3796 €	6688 €	< 0,001
*Erlös*				
(Intercept)	8647 €	7775 €	9519 €	< 0,001
ASA > 2	1124 €	−106 €	2355 €	0,073
Anämie bei Aufnahme	1473 €	78 €	2868 €	0,039
Infektion der Hüftgelenkprothese	3567 €	2228 €	4907 €	< 0,001
Chronisches Nierenversagen	1632 €	−79 €	3343 €	0,061
Perioperative Transfusion	2814 €	1578 €	4050 €	< 0,001
*Deckung*				
(Intercept)	3700 €	2061 €	5339 €	< 0,001
Alter	−37 €	−61 €	−12 €	0,003
Anämie bei Aufnahme	−995 €	−1679 €	−312 €	0,004
Infektion der Hüftgelenkprothese	−1924 €	−2597 €	−1251 €	< 0,001
Diabetes	−607 €	−1377 €	164 €	0,123
Herzinsuffizienz	770 €	−154 €	1693 €	0,102
Perioperative Transfusion	−2455 €	−3076 €	−1833 €	< 0,001

Die jeweils eingeschlossenen Variablen wurden mittels AIC-Selektion ermittelt. Daher sind nicht in allen Regressionen dieselben Variablen enthalten

Neben präoperativer Anämie und perioperativer Transfusion erhöhten eine Infektion der Hüftgelenkprothese als Operationsindikation und das Lebensalter signifikant die Fallkosten und Erlöse, bei negativer Falldeckung (Tab. 3). Komorbiditäten hatten keinen signifikanten Einfluss auf Kosten, Erlöse oder Falldeckung.

#### Interaktionsanalyse

In Abhängigkeit von der Anzahl der Transfusionen stiegen bei anämischen und nichtanämischen Patienten die mittleren Fallkosten.

**Figure 2:**
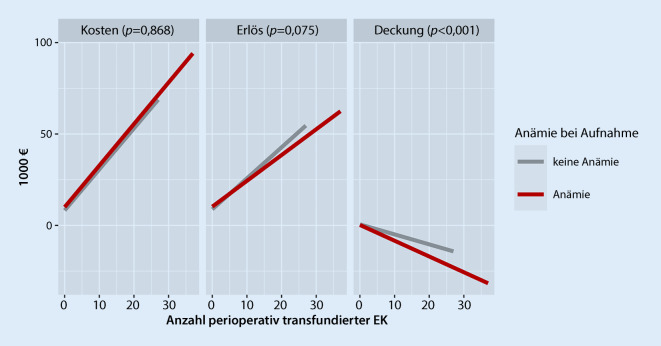

Wie in Abb. 2 dargestellt, war hinsichtlich der Fallkosten keine Interaktion von präoperativer Anämie und der Anzahl perioperativ transfundierter EK nachweisbar (*p* = 0,868). Bezüglich des Fallerlöses zeigte sich eine schwache, jedoch nicht signifikante Interaktion (*p* = 0,075): Bei mehr perioperativen EK-Transfusionen stieg der durchschnittliche Erlös bei präoperativ anämischen Patienten in geringerem Ausmaß als bei nichtanämischen an.

Eine signifikante Interaktion schließlich fand sich bei der Falldeckung (*p* < 0,001). Wurden perioperativ keine EK verabreicht, war die Deckung für anämische und nichtanämische Patienten beinahe identisch. Mit Zunahme der Zahl an EK-Transfusionen nahm die Unterdeckung, also das Defizit, für anämische Patienten stärker zu als für nichtanämische. Auch dieses Ergebnis ist unter Einbezug intraoperativer Transfusionen reproduzierbar (Daten im Zusatzmaterial online, Box am Anfang des Artikels).

## Diskussion

Die Ergebnisse unserer Analyse zeigen zum ersten Mal für das deutsche DRG-System, dass die präoperative Anämie, die eine perioperative EK-Transfusion zur Folge hat, erhöhte Kosten, Erlöse und eine resultierend defizitäre Falldeckung bei Patienten verursacht, die sich einer HGRO unterziehen, wohingegen diese ohne eine perioperative Transfusion kostendeckend ist.

Ein wichtiger Kostentreiber ist dabei die Liegedauer, die bezüglich des Gesamtaufenthalts und der intensivstationären Behandlung bei präoperativer Anämie und perioperativer Transfusion signifikant verlängert war. Dadurch erklärt sich in der Folge die niedrigere Falldeckung. Der mit jedem transfundierten EK steigende Fallerlös wurde dabei nicht durch das Vorliegen einer Anämie beeinflusst.

Schätzungen bezüglich der Kosten von EK-Transfusionen, in Europa zwischen etwa 150 € [12] und 440 € [1], berücksichtigen ausschließlich die Kosten der stationären Behandlung und der Produktion, Bereitstellung, intrahospitalen Logistik und Transfusion von EK, während unsere Arbeit, basierend auf einer Kosten- und Erlösanalyse, eine exakte Berechnung der defizitären Falldeckung präoperativ anämischer Patienten in Abhängigkeit von der Anzahl perioperativer Transfusionen zeigt. Eine vergleichbare Analyse für eine spezifische Komorbidität (die präoperative Anämie) in einem hinsichtlich des untersuchten Eingriffs einheitlichen Kollektiv wurde nach Kenntnis der Autoren bislang nicht publiziert.

Zur Vermeidung der Mehrkosten für Krankenhaus und Volkswirtschaft könnte es sinnvoll sein, die präoperative Anämie vor elektiven Eingriffen ursachengerecht zu therapieren und Transfusionszahlen zu senken [14], wie dies klinische PBM-Konzepte bzw. die deutsche S3-Leitlinie vorsehen [5]. Wenn auch bei diesen Behandlungskonzepten zunächst Kosten für die Implementierung, Anämiediagnostik und -therapie entstehen, wird eine mittelfristige Amortisation in einer deutschen Kosten-Nutzen-Analyse für möglich gehalten [12], müsste jedoch in einer prospektiven randomisierten und multizentrischen Studie bestätigt werden. Die Schlussfolgerung einer englischen Metaanalyse von 2019, laut der keine Studie eine Kosteneffizienz von PBM nachweisen konnte [18], wurde für ihr Design und Ungenauigkeiten kritisiert [7, 9]. Daten aus Australien und Deutschland von 2021 lassen eine solche Effizienz vermuten [2, 22].

Innerhalb des Analysezeitraums änderten sich die erstatteten Fallwerte. Der Anstieg der Erstattungen betrug beispielsweise für die Fallgruppe I64B („Prothesenwechsel am Hüftgelenk ohne äußerst schwere CC, ohne Eingriff an mehreren Lokalisationen, mit periprothetischer Fraktur“) jährlich etwa 1–3 % [3]. Bis auf einen Inflationsausgleich bleiben die Zahlen also vergleichbar.

Auch wenn die Ergebnisse der vorliegenden Studie eine klare Tendenz aufweisen, sollten folgende Limitationen der Methodik beachtet werden:

- Die analysierten Daten wurden zwar digital ausgelesen, unterliegen jedoch – wie jede Datenbank – einer gewissen Fehleranfälligkeit während der manuellen Eingabe. Da die Datenbankeingabe jedoch ausschließlich durch speziell geschultes Personal (Medizinische Dokumentationsassistent*innen) vorgenommen wurde, sind größere oder systematische Fehleingaben als wenig wahrscheinlich anzusehen.
- Durch Untercodierung könnten ICD-10-Diagnosen in den Patientendaten, die möglicherweise die Verteilung der Basischarakteristika beeinflusst haben, fehlen. Das Hauptaugenmerk dieser Arbeit, nämlich der Einfluss der präoperativen Anämie und perioperativer Transfusionen auf Kosten, Erlös und Falldeckung, bleibt davon unbetroffen.
- Wie in Tab. 1 ersichtlich ist, waren präoperativ anämische Patienten auch signifikant häufiger vorerkrankt. Da sich die Ergebnisse bezüglich Kosten, Erlös und Deckung auch nach einem Nearest Neighbor Propensity Score Matching (nicht angehängt) mit den Kovariaten Alter, Geschlecht, ASA-Score > 2 und Art der Hüftkomplikation – also nach Zuordnung jedes anämischen Patienten zu genau einem nichtanämischen, der diesem in Bezug auf die genannten Kovariaten am meisten ähnelte – replizieren ließen, gehen wir davon aus, dass die präoperative Anämie keinen bloßen Indikator für allgemein schwerer erkrankte Patienten darstellt.
- Die vorliegende Studie berücksichtigt nur Fremdbluttransfusionen, nicht jedoch die Retransfusion von gewaschenem Wundblut mit der maschinellen Autotransfusion. Für die Analyse der Kosten, Erlöse und Deckungen von präoperativer Anämie und perioperativer EK-Transfusion scheint dies vertretbar: In einer Studie an 191 Patienten, die sich einer Lebertransplantation unterzogen, erwiesen sich Fremdbluttransfusionen als stärkster unabhängiger Prädiktor der Gesamtkosten, während die maschinelle Autotransfusion keinen Einfluss auf die Gesamtkosten hatte [19].
- Die lineare Regression diente dem Zweck, Nebenerkrankungen als Störfaktoren auszuschließen. Durch die Begrenzung der Auswahl ist es naturgemäß möglich, dass nicht alle Störfaktoren (Confounder) berücksichtigt wurden.
- Eine evtl. präoperative Anämiebehandlung wurde elektronisch nicht erfasst und konnte somit auch nicht ausgelesen werden. Da eine präoperative Anämietherapie aufgrund einer fehlenden Finanzierung bisher nicht flächendeckend etabliert ist und im Beobachtungszeitraum die deutsche S3-Leitlinie „Präoperative Anämie“ noch nicht publiziert war [5], ist hier von keinem hohen Fehlerpotenzial auszugehen.

## Fazit für die Praxis

Zusammenfassend ist das Ergebnis dieser Arbeit, die als Erste unter den Bedingungen des deutschen DRG-Systems die Kosten, Erlöse und Falldeckung von präoperativer Anämie und Fremdbluttransfusion bei Patienten mit Hüftgelenkprothesenrevisionsoperationen analysiert, dass präoperative Anämie und perioperative Fremdbluttransfusion mit signifikant höheren Kosten und einer niedrigeren Falldeckung assoziiert sind. Patienten mit perioperativer Transfusion wiesen längere Liegedauern und im Median eine negative Falldeckung auf. Die sorgfältige Implementierung eines Konzepts zu Diagnostik und Therapie der präoperativen Anämie zur Vermeidung perioperativer EK-Transfusionen könnte mittelfristig eine Entlastung sowohl für die Krankenhäuser als auch die Krankenkassen erzielen.

## Supplementary Information




