# Supplementary material for: The costs of preoperative anemia in hip joint revision surgery
Source: Anaesthesiologie. 2022 Nov 15;72(1):13–20. [Article in German] doi: 10.1007/s00101-022-01211-x (PMC9852200; doi:10.1007/s00101-022-01211-x)

Zusatzmaterial zum Beitrag **„Die Kosten der präoperativen Anämie bei Hüftgelenksrevisionsoperationen“ von Vorderwülbecke G, Spies C, v. Heymann C et al. (2022) in *Die Anaesthesiologie***
Beitrag und Zusatzmaterial stehen Ihnen auf www.springermedizin.de zur Verfügung. Bitte geben Sie dort den Beitragstitel in die Suche ein.

Kostendaten und Liegedauern in Abhängigkeit von präoperativer Anämie

	**gesamt**	**keine Anämie**	**Anämie**	**p-Wert**
**Alle Patienten**	*N=1187*	*N=833*	*N=354*	
Kosten [€]	9475 [7826;12858]	8948 [7501;11339]	12318 [9027;20044]	<0.001
Erlös [€]	9853 [8514;12304]	9611 [8332;10719]	11788 [8992;16298]	<0.001
Deckung [€]	68 [-2170;1926]	591 [-1441;2103]	-1170 [-4467;1238]	<0.001
Liegedauer [Tage]	11 [9;15]	11 [9;14]	15 [11;23]	<0.001
ITS-Dauer [Tage]	0 [0;8]	0 [0;4]	4 [0;21]	<0.001
**Perioperativ nicht transfundierte Patienten**	*N=622*	*N=525*	*N=97*	
Kosten [€]	8238 [7043;9797]	8152 [6942;9524]	9049 [7747;11425]	<0.001
Erlös [€]	9572 [8029;10109]	8992 [8002;10093]	9816 [8576;12546]	<0.001
Deckung [€]	1060 [-408;2356]	1098 [-333;2357]	520 [-922;2295]	0.142
Liegedauer [Tage]	10 [9;12]	10 [9;12]	11 [9;14]	<0.001
ITS-Dauer [Tage]	0 [0;0]	0 [0;0]	0 [0;4]	0.010
**Perioperativ transfundierte Patienten**	*N=565*	*N=308*	*N=257*	
Kosten [€]	11934 [9332;18235]	11005 [8996;14511]	13978 [9949;22734]	<0.001
Erlös [€]	10779 [8992;13939]	9957 [8576;12700]	12304 [9850;16796]	<0.001
Deckung [€]	-1464 [-4407;639]	-1056 [-3242;823]	-2366 [-6631;283]	<0.001
Liegedauer [Tage]	14 [11;21]	13 [10;16]	16 [12;27]	<0.001
ITS-Dauer [Tage]	5 [0;19]	3 [0;15]	8 [0;29]	<0.001

Kostendaten und Liegedauern in Abhängigkeit von Transfusion insgesamt

	**gesamt**	**keine Transfusion**	**Transfusion**	**p-Wert**
**Alle Patienten**	*N=1187*	*N=559*	*N=628*	
Kosten [€]	9475 [7826;12858]	8147 [6937;9513]	11696 [9195;17476]	<0.001
Erlös [€]	9853 [8514;12304]	8992 [8015;10093]	10779 [8921;13847]	<0.001
Deckung [€]	68 [-2170;1926]	1122 [-332;2372]	-1225 [-4051;832]	<0.001
Liegedauer [Tage]	11 [9;15]	10 [9;12]	14 [11;19]	<0.001
ITS-Dauer [Tage]	0 [0;8]	0 [0;0]	5 [0;18]	<0.001
**Präoperativ anäme Patienten**	*N=354*	*N=63*	*N=291*	
Kosten [€]	12318 [9027;20044]	8829 [7307;10428]	13513 [9618;21767]	<0.001
Erlös [€]	11788 [8992;16298]	9572 [8502;11917]	12120 [9813;16740]	<0.001
Deckung [€]	-1170 [-4467;1238]	516 [-908;2203]	-1729 [-6026;724]	<0.001
Liegedauer [Tage]	15 [11;23]	10 [9;14]	16 [12;25]	<0.001

ITS-Dauer [Tage]	4 [0;21]	0 [0;0]	7 [0;26]	<0.001
**Präoperativ nicht-anäme Patienten**	*N=833*	*N=496*	*N=337*	
Kosten [€]	8948 [7501;11339]	8086 [6832;9406]	10905 [8948;14309]	<0.001
Erlös [€]	9611 [8332;10719]	8992 [8002;10093]	10093 [8576;12700]	<0.001
Deckung [€]	591 [-1441;2103]	1196 [-233;2393]	-1003 [-3240;935]	<0.001
Liegedauer [Tage]	11 [9;14]	10 [9;12]	12 [10;16]	<0.001
ITS-Dauer [Tage]	0 [0;4]	0 [0;0]	3 [0;14]	<0.001

Graphische Darstellung transfundierter Erythrozytenkonzentraten

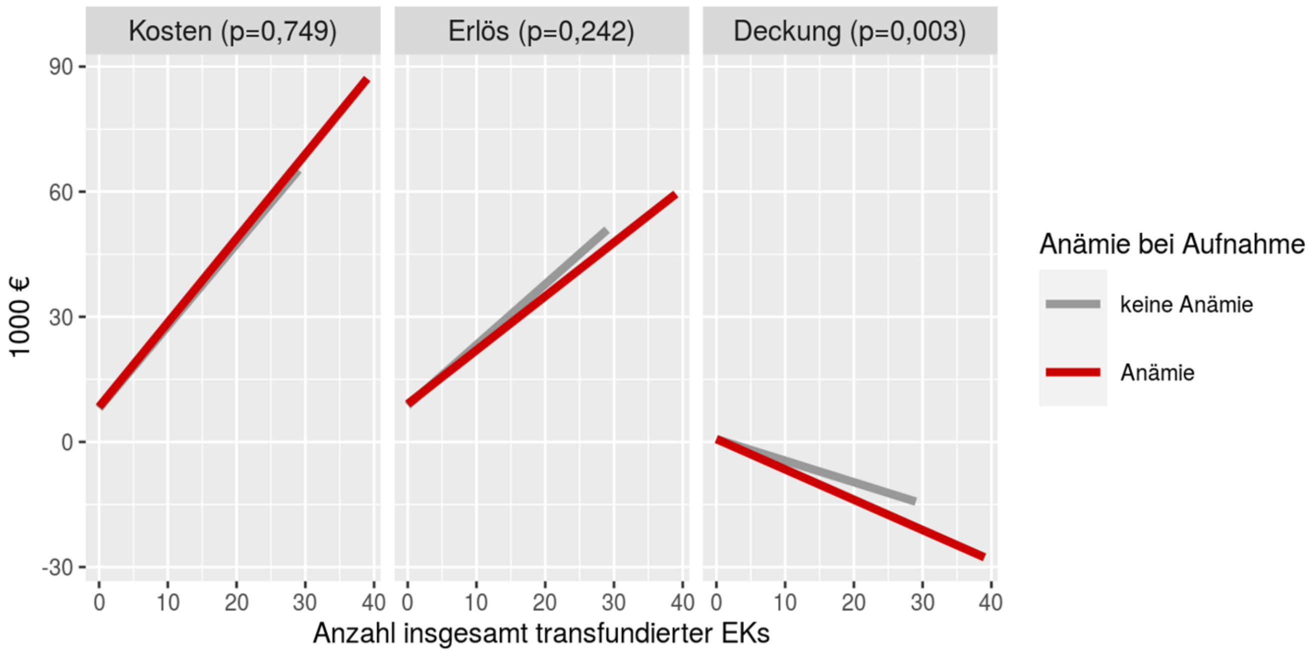

Supplement: Supplementary file 1 [file 101_2022_1211_MOESM1_ESM.pdf]
